# Evaluation of applicability of the Sartorius Airport MD8 sampler for detection of *Bacillus* endospores in indoor air

**DOI:** 10.1007/s10661-012-2807-6

**Published:** 2012-08-10

**Authors:** Rafał Lewandowski, Krystyna Kozłowska, Małgorzata Szpakowska, Elżbieta A. Trafny

**Affiliations:** Department of Microbiology, Military Institute of Hygiene and Epidemiology, Kozielska 4, 01-163 Warsaw, Poland

**Keywords:** *Bacillus atrophaeus*, Sartorius AirPort MD8 sampler, Bioaerosol, Indoor air

## Abstract

This study was designed to evaluate the measuring range and lowest limit of detection of *Bacillus* endospores in the ambient room air when the Sartorius MD8 sampler, and two different culture methods for bacterial enumeration were used. Different concentrations of bioaerosol were generated inside the test chamber filled with either the high-efficiency particulate air (HEPA)-filtered air or with the ambient room air. The detection of endospores in the HEPA-filtered air was achievable: (1) when they were aerosolized at a concentration above 7.56 × 10^3^ CFU/m^3^ and analyzed with spread plate method, and (2) when they were aerosolized at a concentration above 4.00 × 10^2^ CFU/m^3^ and analyzed with pour plate method. The detection of endospores in the ambient room air was possible: (1) when they were aerosolized at a concentration above 9.1 × 10^3^ CFU/m^3^ and analyzed with spread plate method, and (2) when they were aerosolized at a concentration above 5.6 × 10^2^ CFU/m^3^ and analyzed with pour plate method. The microorganisms present in the ambient room air interfere with precise quantification of *Bacillus* endospores when their concentration is relatively low. The results of this study may be helpful in critical assessment of the results obtained from monitoring the air for bacterial endospores.

## Introduction

Airborne microorganisms, widely found in the natural environment, may pose a significant risk to human health and cause infectious and/or noninfectious respiratory problems. Moreover, these microorganisms can also be used as weapons, as has been observed during anthrax bioterrorist incidents in October 2001 (CDC 2001; Weis et al. 2002). The aerosolized *Bacillus* endospores are more dangerous than other airborne biowarfare agents because they are not susceptible to environmental stresses and preserve their viability in the air better than the vegetative form of the bacteria. Effective air sampling of endospores is an important element of an early warning/rapid response system against airborne biological threat agents. Impaction, impingement, and filtration are currently available methods for efficient sampling of airborne microorganisms. To perform this task, one can choose among several air samplers that are commercially available. The most literature data regarding efficiency and operational range of the samplers have been focused on two impaction-based samplers (a six-stage Andersen impactor and a MAS-100 sampler) (Mainelis and Tabayoyong 2010; Raisi et al. 2010), two liquid-type impingers (an AGI-30 sampler and a BioSampler) (Albrecht et al. 2007; Rule et al. 2009), and two-filter based samplers (a 37-mm cassette sampler and a button sampler) (Lee et al. 2006; Hwang et al. 2010). The analysis of bioaerosol samples relies mainly on the culture-based methods that provide a number of culturable bacteria in the air sample (Caruana 2011).

The selection of suitable bioaerosol sampler for detection of unknown airborne microorganisms and assessment of contamination level is not an easy task. The latter could be influenced by the species of microorganisms that are sought in the sample (vegetative cells versus endospores) and the lowest detection limit of both the bioaerosol sampling and the analytical method. In the contaminated indoor environment, a filtration is an easy-to-use method to collect bioaerosols. However, dehydration of the vegetative cells during sampling may reduce the bacterial survival rates; thus, the reliable estimation of the number of viable bacteria is not possible (Mandal and Brandl 2011). The gelatin membrane filters have been used for bioaerosol sampling in order to decrease the desiccation of bacterial cells (Wu et al. 2010). Moreover, gelatin membrane filters have high filtration efficiency rates (99.9995 %) (Parks et al. 1996). The gelatin membrane filters (3 μm pore size) can be used in combination with the Sartorius Airport MD8 sampler to collect microorganisms from the air. Although the Sartorius Airport MD8 sampler have already been employed in several studies for monitoring the microbiological quality of the air (Engelhart et al. 2007: Zhao et al. 2010), there is still a lack of data on its measuring range and analytical sensitivity (minimum detectability of bacterial endospores) in combination with culture-based analysis of the ambient air samples.

The objective of this study was to estimate the measuring range and the lowest limit of detection of *Bacillus atrophaeus* endospores (used as a surrogate for *Bacillus anthracis* endospores) in the ambient room air sampled by the Airport MD8 device equipped with gelatin membrane filters.

## Materials and methods

### *Bacillus* endospores

The bacterial strain used in this study was *B. atrophaeus* ATCC 9372. The *B. atrophaeus* endospores were obtained from cultures grown on a solid 2×SG sporulation medium for 5 days at temperature of 35 °C, followed by culture at room temperature. The endospores were purified as described in Lewandowski et al. (2010). Finally, endospores were kept in a solution of ethanol in water at 4 °C. The viability of the endospores was routinely checked every 3 months. The suspension of *B. atrophaeus* endospores for aerosol generation was prepared in 96 % ethanol a day before each experiment and kept in refrigerator until use. The optical density of the spore suspension was adjusted to 1.0 when it was read at a wavelength of 600 nm with a SP6-500 UV spectrophotometer (Pye Unicam Ltd., Cambridge, UK). The number of the culturable endospores in the suspension was estimated by dilution and plating onto Luria–Bertani (LB) (Sigma-Aldrich, St. Louis, MO, USA) agar plates.

### Bioaerosol experimental system

Bioaerosols were generated in a test chamber (model 830-ABB/Sp with 800-HEPA/D; Plas-Labs, Inc., Lansing, MI, USA). The chamber had an interior volume of 0.5 m^3^ and was placed in a laboratory room with exhaust ventilation. The *B. atrophaeus* endospores were aerosolized inside the chamber filled with a high-efficiency particulate air (HEPA)-filtered air or with an ambient room air that was pumped into the chamber. Bioaerosols were generated by a compressed-air nebulizer Monsun 2 MP2 equipped with a RF6 head (Medbryt, Warsaw, Poland). The nebulizer was placed outside the chamber and was connected with tubing with the RF6 head through a valve and a HEPA filter (with a diameter of 5.5 cm). The RF6 head was placed 0.65 m above the bottom surface of the chamber. Ten-mililiter samples of a *B. atrophaeus* spore suspension in 96 % ethyl alcohol at concentrations ranging from 2.0 ± 0.70 × 10^1^ to 4.03 ± 1.71 × 10^7^/ml were aerosolized inside the chamber at 3.2 × 10^5^ Pa pressure, an airflow rate of 15.5 l/min, and a liquid generation rate of 0.48 ml/min. According to the manufacturer, the nebulizer generates airborne particles with a mass median aerodynamic diameter of 1.4 μm. The ambient room air was pumped into the chamber by a compressed-air nebulizer D-1 (Medbryt, Warsaw, Poland). Four VDC-001 Life-Desktop USB fans (0.14 m diameter, Veho, Eastleigh, Hampshire, UK) were used to stir air inside the chamber. The fans were situated along the diagonals of the chamber, 0.20 m above the bottom of the chamber. This arrangement was used to reduce gravitational settling of the aerosolized *B. atrophaeus* endospores during experiments. After each trial, the chamber was decontaminated with PeraSafe (Antec International DuPont, Sudbury, Suffolk, UK) and rinsed with water. Before each experiment, a UVC lamp (Puritec LPS9; OSRAM, GmgH, Augsburg, Germany) was switched on inside the chamber for 1 h. Then, interior air was exchanged through a HEPA filtration system for 30 min. All trials were conducted at room temperature (20–23 °C).

### Microbiological air sampling and analysis

Airborne microorganisms were sampled using four air samplers: an Airport MD8 gelatin filter sampler (Sartorius AG, Göttingen, Germany), a SKC BioSampler liquid impinger model 225-9595 (SKC Inc., Eighty Four, PA, USA) and two impaction-based air samplers: an Andersen six-stage impactor model TE-10-800 (Tisch-Environmental Inc., Cleves, OH, USA) and a MAS-100 single-stage impactor (Merck, Darmstadt, Germany). The samplers were placed outside the chamber and were connected with tubing with a valve placed inside the chamber. This arrangement ensured multiple sampling of bioaerosols during the same experiment using different air samplers.

The Sartorius Airport MD8 sampler used in our experiments was equipped with a 1.5-m length, 38-mm diameter flexible plastic hose connected with a filter head containing an 80-mm diameter gelatin membrane filter (a pore size of 3 μm, Sartorius Stedim Biotech GmbH, Göttingen, Germany). The sampler was operated at a flow rate of 50 l/min, and the volumes of air sampled were equal to 0.1, 0.5, and 1.0 m^3^. After air sampling, filters were removed from the sampling head using sterile forceps and were dissolved in 30 ml of 0.01 M phosphate-buffered saline (PBS) and shaken in a temperature-controlled water bath for 10 min at 35 °C. The suspensions were serially diluted in sterile deionized water (SDW) in five repetitions and cultivated onto LB agar using the spread plate or the pour plate method. In a spread plate method, 0.1 ml of the serially diluted bacterial suspensions were pipetted onto the surface of LB agar and evenly distributed with spreaders. In a pour plate method, 1.0 ml of the serially diluted bacterial suspensions were pipetted in five repetitions into Petri dishes and mixed with 14 ml of molten LB agar. Plates were placed on a flat surface for about 10 min to allow the agar to completely gel. The spread plates and the pour plates were incubated at 35 °C for 7 days. The colonies were counted, and the final results were expressed as CFU per cubic meter of air (CFU/m^3^).

The SKC BioSampler that contained 15 ml of PBS was connected to a BioLite air-sampling vacuum pump, model 228-9610 (SKC Inc., Eighty Four, PA, USA). Sampling was carried out at airflow of 12.5 l/min, and the volume of air sampled was equal to 0.125 m^3^. Before each experiment, the impinger was calibrated with rotameter model EK-5SR-H (Kytola Instruments, Finland) to the recommended flow rate. After air sampling, the suspensions were serially diluted in SDW prior to inoculation onto LB agar using the spread plate method. The plates were incubated at 35 °C for 7 days. The colonies were counted, and the final results were expressed as CFU/m^3^.

The Andersen impactor was joined to vacuum pump model S37MYHCD-1454 (Emerson Motor Technologies, St. Louis, MO, USA). The flow rate was adjusted to 28.3 l/min using rotameter model EK-5SR-H. The volumes of the samples collected were 0.1, 0.5, or 1.0 m^3^, respectively. After each trial, the impactor was sanitized with 70 % ethyl alcohol. All plates (LB agar) were incubated at 35 °C for 7 days. The colony counts were reported by the positive-hole method according to Andersen (1958). The results were expressed as total CFU enumerated from all six stages and calculated per cubic meter of air (CFU/m^3^).

The MAS100 sampler was operated at a flow rate of 100 l/min, and the volumes of air sampled were equal to 0.1, 0.5, or 1.0 m^3^. After each trial, the sampler was sanitized with 70 % ethyl alcohol. All plates were incubated at 35 °C for 7 days. The numbers of colonies growing on LB agar were reported using a positive holes correction table according to manufactures’ instruction. The results were expressed as total CFU and calculated per cubic meter of the air collected.

## Statistical analyses

Values are expressed as mean ± standard error of the mean (SEM). The comparison between the number of viable bacteria in the air samples enumerated using the spread plate method and the pour plate method was submitted to Student’s *t* test. All values were considered statistically significant at *P ≤* 0.05 (Microsoft Office Excel for Windows). Linear regression lines were automatically drawn using a software graphics package (SigmaPlot; Jandel, San Rafael, CA, USA) for the number of the aerosolized *B. atrophaeus* spores and the number of viable bacteria recovered from the test chamber filled with either the HEPA filtered air or with the ambient room air.

## Results

### Performance of the air samplers for quantification of *Bacillus* endospores

**Fig. 1:**
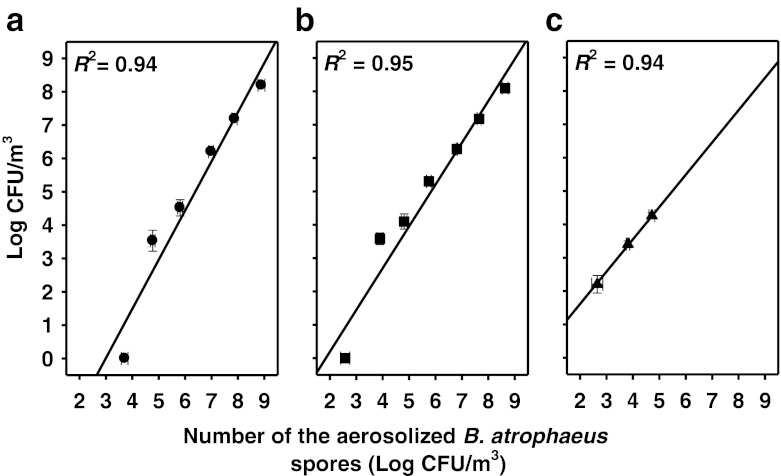
Relationship between the numbers of *B. atrophaeus* spores collected using BioSampler (**a**), Airport MD8 (**b**), and six-stage Andersen impactor (**c**) and the total number of spores aerosolized within the chamber filled with the HEPA-filtered air. Data are shown as the mean log values (±SD) per cubic meter of air. Linear regression coefficients (*R* ^2^) are shown in the upper right corner of each graph

In a first step of experiments, in order to compare the measuring range of the three devices widely used for air sampling, we collected the bioaerosols of various concentrations [in a range from 3.43 × 10^2^ (±1.68 × 10^2^) to 8.05 × 10^8^ (±2.32 × 10^8^) CFU/m^3^] generated in a test chamber filled with a HEPA-filtered air. The number of *B. atrophaeus* endospores in these bioaerosol samples collected using the Airport MD8 sampler, the SKC BioSampler, and the Andersen six-stage impactor were dependent on the concentration of the endospores aerosolized within the chamber. As shown in Fig. 1, the linear regression coefficient (*R* ^2^) for the data obtained with the BioSampler was 0.94, when the number of the endospores aerosolized was in the range from 6.01 × 10^4^ (±1.29 × 10^4^) to 8.05 × 10^8^ (±2.32 × 10^8^) CFU/m^3^ of air. A similar relationship (*R* ^2^ = 0.95) was observed when the number of total CFU aerosolized inside the chamber [from 7.56 × 10^3^ (±1.58 × 10^3^) to 4.47 × 10^8^ (±1.03 × 10^8^) CFU/m^3^ of air] was plotted versus the concentration of the endospores taken with the Airport MD8 sampler. A high regression coefficient (*R* ^2^ = 0.94) was also observed when bioaerosol samples were taken using the Andersen six-stage impactor; however, only when the number of aerosolized endospores was in the range from 4.80 × 10^2^ (±1.79 × 10^2^) to 5.31 × 10^4^ (±1.31 × 10^4^) CFU/m^3^ of air. Thus, the measuring range of the Andersen impactor was observed in the lowest concentration range of the bacteria examined in this study.

### Microbiological examination of the ambient room air

**Fig. 2:**
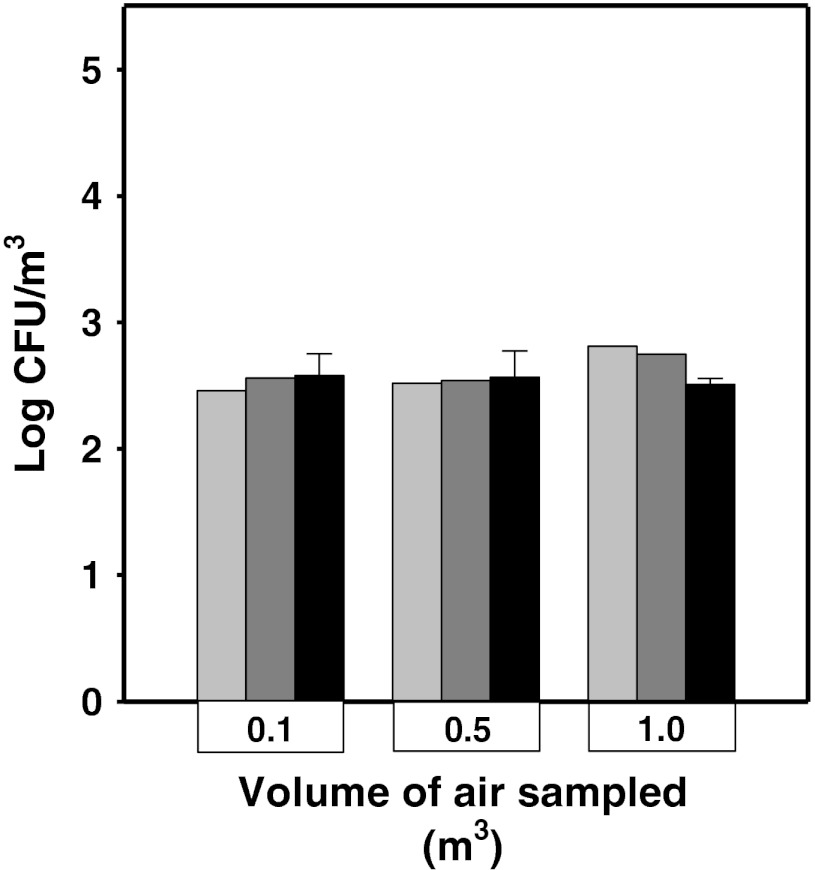
Comparison of the number of culturable microorganisms in the samples of ambient air collected using the following air samplers: MAS100 (*light gray bars*), six-stage Andersen impactor (*dark gray bars*), and Airport MD8 (*black bars*) in the laboratory room. The bars are shown as the log values (*light or dark gray bars*) or as the mean log values (±SD) (*black bars*)

Every time before start of the aerosolization of *B. atrophaeus* endospores in the test chamber filled with the ambient room air, the air samples were taken outside the chamber for microbiological analysis. A total of three samples were collected per sampling event with use of the three air samplers (Airport MD8, the six-stage Andersen impactor, and MAS-100), as well as three different volumes of air samples (0.1, 0.5, and 1.0 m^3^) were taken. As it was shown in Fig. 2, the mean number of culturable microorganisms collected using the Airport MD8 sampler was similar when the volume of the air sampled were 0.1, 0.5, and 1.0 m^3^, and was equal to 4.0 × 10^2^ (±1.73 × 10^2^), 4.02 × 10^2^ (±1.84 × 10^2^), and 3.24 × 10^2^ (±3.91 × 10^2^) CFU/m^3^, respectively. The number of microorganisms recovered with use of MAS100 was comparable to the number of microorganisms collected with the six-stage Andersen.

### Recovery of endospores aerosolized within the chamber in the ambient air or in the HEPA-filtered air

The Airport MD8 sampler was chosen for further studies based on both its measuring range and a high efficiency for trapping of culturable microorganisms from both the relatively small volume (0.1 m^3^) and a large volume (1.0 m^3^) of ambient air. *B. atrophaeus* endospores were aerosolized within an experimental chamber at five different concentrations. Two approaches to microbial analysis of the air sampled were undertaken with regard to culture of the viable endospores: a spread plate technique and a pour plate technique.

The average recovery of endospores from the bioaerosol samples taken from the chamber filled with either the HEPA-filtered air or the ambient air using the Airport MD8 sampler was similar (no significant differences in *t* tests, *P* ≥ 0.05), when the air contamination was relatively high, i.e., in the range from approximately 6 × 10^5^ to 8 × 10^3^ CFU/m^3^, and from 6 × 10^5^ to 9 × 10^3^ CFU/m^3^, respectively (Table 1). When the level of the air contamination with *Bacillus* endospores was relatively low (below 10^3^ CFU/m^3^), then virtually no endospores were detected in the HEPA-filtered air with the use of spread plate technique, although the pour plate technique allowed to detect 7 × 10^2^ endospores in the 1.0 m^3^ of the air samples collected with the sampler.

**Table 1:** The concentration of aerosolized *B. atrophaeus* spores and the number of viable bacteria recovered from the test chamber filled with either the HEPA filtered air or with the ambient room air using the Airport MD8 sampler

Bioaerosol generated in	Mean (CFU/m^3^)			*P* value^a^
	The aerosolized *B. atrophaeus* spores	The recovered viable bacteria		
		Spread plate method	Pour plate method	
The HEPA-filtered air	5.70 × 10^5^ ± 1.15 × 10^5^	2.03 × 10^5^ ± 3.77 × 10^4^	2.05 × 10^5^ ± 2.85 × 10^4^	0.90
	6.68 × 10^4^ ± 1.65 × 10^4^	1.38 × 10^4^ ± 6.22 × 10^3^	1.25 × 10^4^ ± 3.00 × 10^3^	0.60
	7.56 × 10^3^ ± 1.58 × 10^3^	4.00 × 10^3^ ± 1.73 × 10^3^	5.33 × 10^3^ ± 2.87 × 10^2^	0.36
	4.00 × 10^2^ ± 1.41 × 10^2^	0.00 ± 0.00	6.75 × 10^2^ ± 2.87 × 10^2^	^-^
	4.00 × 10^1b^	0.00 ± 0.00	0.00 ± 0.00	^-^
The ambient air	6.43 × 10^5^ ± 9.62 × 10^4^	3.49 × 10^5^ ± 6.02 × 10^4^	3.27 × 10^5^ ± 4.73 × 10^4^	0.58
	6.99 × 10^4^ ± 1.58 × 10^4^	2.76 × 10^4^ ± 8.32 × 10^3^	3.50 × 10^4^ ± 6.93 × 10^3^	0.19
	9.10 × 10^3^ ± 2.00 × 10^3^	6.38 × 10^3^ ± 3.37 × 10^3^	6.30 × 10^3^ ± 2.18 × 10^3^	0.97
	5.60 × 10^2^ ± 3.58 × 10^2^	3.00 × 10^3^ ± 0.00	6.63 × 10^3^ ± 1.60 × 10^3^	0.01
	5.60 × 10^1b^	0.00 ± 0.00	7.80 × 10^2^ ± 6.22 × 10^2^	^-^

A volume of the sample collected was 100 l of air and the samples were taken immediately after the bioaerosol generation

^a^ *P* values comparing the spread plate method with the pour plate method (Student’s *t* tests)

^b^The number calculated by dividing the concentration of the bacterial suspension presented in the row above by 10 to get the most probable concentration of the endospores in the test chamber

When similar analysis was performed in the ambient room air that was pumped into the experimental chamber, the growth of the bacteria was observed on both the spread and pour plates. However, when the number of the aerosolized endospores was below 10^2^ CFU/m^3^ no microorganisms were detected on spread plates, but an average of 2.6 (±2.07) bacterial colonies were observed in pour plates. In these experiments, the colonies in pour plates were not identified as *B. atrophaeus*, when the number of the endospores in bioaerosol was below 10^3^ CFU/m^3^. Surprisingly, the microorganisms naturally occurring in the ambient air were detected neither on spread plates nor in pour plates in similar experiments, when the densities of endospores in the aerosols were in range from 10^3^ to 10^5^ or from 10^2^ to 10^5^ CFU/m^3^, respectively.

Therefore, the detection of endospores in the 1.0 m^3^ of the ambient air was achievable: (1) when the endospores were aerosolized at a concentration above 9.1 × 10^3^ CFU/m^3^ and analyzed with the spread plate method, and (2) when endospores were aerosolized at a concentration above 5.6 × 10^2^ CFU/m^3^ and analyzed with the pour plate method. Thus, using the pour plate method lowered the concentration of the detectable endospores in the ambient air samples more than ten times (16 times).

**Fig. 3:**
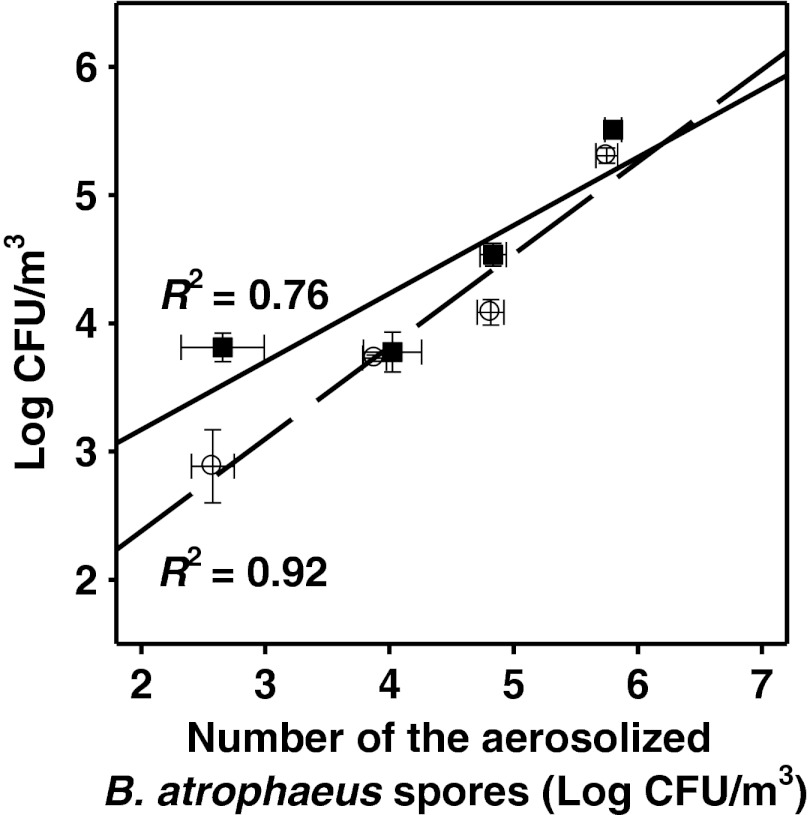
The correlation between the numbers of *B. atrophaeus* spores collected using Airport MD8 sampler and the total number of spores aerosolized within the test chamber filled either with the ambient air (*square*) or with the HEPA-filtered air (*circle*). The culturable bacteria were enumerated using the pour plate method. Data are shown as the mean log values (±SD) per cubic meter of air. Linear regression coefficients (*R* ^2^) are shown beside the each graph

When the Airport MD8 sampler was used to collect bioaerosol samples and the pour plate method was employed for analysis of the number of culturable bacteria in the samples, a high correlation (*R* ^2^ = 0.92) was observed between the number of aerosolized endospores and the number of the bacteria detected (Fig. 3). However, it holds true only when the experimental chamber was filled with HEPA-filtered air. When the sampling and analysis was made in the ambient air, the linear regression coefficient was significantly lower (*R* ^2^ = 0.76). Therefore, the presence of other organisms and dust particles in ambient air might interfere with *Bacillus* endospores collection in gelatin membrane filters and their enumeration with microbial culture techniques.

## Discussion

Many commercially available air samplers based on different physical principles have been used for microbiological examination of indoor air. When quantification of viable microorganisms is an objective of air sampling, the impingement, impaction or filtration with gelatin membrane filters are preferable methods for collection of endospores and vegetative bacterial cells (Fabian et al. 2005).

In order to choose an appropriate air sampler to collect the air contaminated by natural or intentional release of highly infectious biological agents, one should consider whether the sampler allows for linear measurement of the concentration of microorganisms in the air samples. In preliminary studies, determination of linear range of bacterial detection with the sampler should be carried out using test chambers under experimental conditions.

Also at the beginning of experimental work, the use of the culture methods is sufficient to enumerate viable bacteria; however, one should bear in mind that for enumeration of all viable organisms (together with viable but unculturable bacteria) in air samples the other methods should also be employed (epifluorescent or molecular methods) (Deloge-Abarkan et al. 2007). In this study, the results of the preliminary experiments are presented. We used endopores of *B. atrophaeus* as a surrogate of *B. anthracis* endospores, in accordance with other studies (Burke et al. 2004; Laflamme et al. 2004; Burton et al. 2005; Lee et al. 2008). Other *Bacillus* species might also be employed for this purpose, including *B. subtilis*, *B. thuringiensis*, *B. mycoides*, and *B. megaterium* species. The different criteria for selection of an appropriate surrogate for *B. anthracis* endospores have been already proposed; among them, a risk of use, genetic and morphological similarity to *B. anthracis*, and response to various chemical and environmental challenges seemed to be most important (Greenberg et al. 2010). An aerodynamic diameter of *B. atrophaeus* endospore is about 0.9 μm and is slightly smaller when compared to *B. anthracis* endospores (Burton et al. 2007; Carrera et al. 2006). *B. atrophaeus* is considered as nonpathogenic and is classified as a BSL-1 organism. Thus, may be safely used in bioaerosol generation and investigation attempts.

The endospores of *B. atrophaeus* were aerosolized in the experimental chamber and the number of the recovered endospores assayed by two methods of bacterial culture: the spread and the pour plate methods. However, in these studies, we also wondered whether aerosolization of endospores in the test chamber, which was filled with the ambient air instead of the HEPA-filtered air, would change the characteristics of the air samples collected and, therefore, affect *Bacillus* endospores detection.

In the first experiments, we compared the measuring range of the three commercially available samplers used to collect endospores from the test chamber filled with the HEPA-filtered air. The bioaerosol was generated at various concentrations. The spread plate method was used for enumeration of microorganisms because of its simplicity. The results showed that the measurement using the Airport MD8 sampler was possible in a wider range of endospore concentrations (from 10^3^ to 10^8^ CFU/m^3^), than using the BioSampler (from 10^4^ to 10^8^ CFU/m^3^). In contrast, the measurement using the six-stage Andersen impactor was efficient when endospore concentrations were from 10^2^ to 10^4^ CFU/m^3^. Similar results have already been shown by Laitinen et al. (1992), who showed a linear relationship over the bacteria concentration range from 10^2^ to 10^5^ CFU/m^3^, when the air samples were taken using the Andersen six-stage impactor. The effective concentration range for the majority of agar-plate impactors is usually considered to be below 10^4^ CFU/m^3^ (Jensen and Schafer 1998).

In our study, for quantification of culturable microorganisms that naturally occur in the ambient air, the two agar-plate impactors (the Andersen six-stage impactor and the MAS-100 sampler) were used because the concentration of microorganisms in the air was anticipated to be relatively low. For comparison purposes, the Sartorius Airport MD8 sampler was also employed in these experiments, and a more sensitive technique of bacteria enumeration—the pour plate technique—was used. The results obtained in these experiments have shown that five- or tenfold increase in volume of the air samples collected (due to increase in sampling time at the same velocity of air flow) did not result in an increase of the mean number of the culturable microorganisms. Similar results have already been reported by Durand et al. (2002) who used filtration method of air sampling at the composting facilities. The authors have demonstrated that increase in sampling time up to 6 h at a stable air flow of 2 l/min did not affect the mean number of culturable bacteria in 1 m^3^ of the air that contained bacteria at the relatively constant concentration in a period of time when the air samples were collected.

In this work, a comparison between the number of the bacteria collected using the Airport MD8 sampler (and grown in pour plates) and the number of the bacteria impacted on agar plates using the MAS 100 sampler and the Andersen apparatus has shown that there were no significant differences between sampling method and the samplers when the air was collected in volumes up to 1 m^3^. Thus, sampling of the ambient air with any of the samplers used provides the same results on the air pollution by microorganisms. These results confirmed the previous reports by Engelhart et al. (2007) who observed comparable performance of both Satrorius MD8 Airport and Merck MAS-100 in air sampling of *Aspergillus fumigatus* and other thermotolerant fungi. The results of these experiments allowed to estimate the microbial quality of the ambient air used to fill the test chamber during further experiments in this study.

In these experiments, the Sartorius MD8 apparatus was used for estimation of the recovery of the aerosolized *B. atrophaeus* endospores in the test chamber filled with the ambient air. At the same conditions, the recovery of the aerosolized *Bacillus* endospores in the HEPA-filtered air was also measured. We noticed that when a concentration of *Bacillus* endospores per cubic meter of the ambient air was below of 5.6 × 10^2^, no *B. atrophaeus* colonies in pour plates have been observed—only the colonies of other bacterial and fungal species. At and above this concentration of endospores in the aerosol generated in the ambient air, no colonies of other microorganisms but *B. atrophaeus* were observed. The lowest number of *B. atrophaeus* colonies observed in pour plates was 16.2 (±3.56), and this value corresponded to 6.63 × 10^3^ endospores in 1 m^3^ of the ambient room air. In the same conditions, but when the endospores were aerosolized in the HEPA-filtered air the lowest number of *B. atrophaeus* colonies in pour plates was 2.25 (±0.96), and it was equal to 6.75 × 10^2^ CFU when expressed per cubic meter of air.

Although a lot of information is available on using the Sartorius MD8 sampler for estimation of outdoor or indoor air pollution in different environments, there is a lack of data concerning use of the sampler for quantification of *Bacillus* endospores aerosolized in the ambient air at different concentrations. Other researchers have shown that the observed limits of detection of the vegetative bacteria from other species were similar to our observations. Landman et al. (2004) have observed the limit of detection of *Enteroccocus faecalis* equal to 7.94 × 10^3^ CFU/m^3^ of air collected using the Sartorius MD8 sampler when bacteria were aerosolized in an empty isolator (with a volume of 1.3 m^3^). In a study performed by Hagesawa et al. (2011), the mean number of *Staphylococcus epidermidis* cells collected using the Sartorius MD8 sampler and estimated with the pour plate method was above 10^4^ CFU/m^3^, when the concentration of aerosolized bacteria was 1.3 × 10^5^ CFU/m^3^. Unfortunately, in these studies, *B. atrophaeus* endospores were not used, and the bacteria were aerosolized in the experimental chambers filled with the HEPA-filtered air only.

Recently, Estill et al. (2011) have described the aerosolization of *B. anthracis* Sterne endospores at relatively low concentrations (from 1 × 10^3^ to 1.7 × 10^4^ endospores/m^3^) in a chamber filled with the HEPA-filtered air. One of the concentrations used for the aerosol generation was 9.1 × 10^3^ particles/m^3^ (as measured using APS), which corresponded to 2.28 × 10^3^ CFU/m^3^ (based on the estimation of 0.22−0.25 CFU/particle). In these experimental conditions, they have observed that the mean air concentration of endospores was equal to 1.5 × 10^3^ CFU/m^3^ when gelatin filters were used as one of the sampling methods, and the air samples were analyzed by plating. Similar results have been obtained in our study when *B. atrophaeus* endospores were aerosolized in the HEPA-filtered air at a concentration of 7.56 × 10^3^ CFU/m^3^. Then, the mean concentration of the endospores recovered using the gelatin filters was equal to 4.00 × 10^3^ CFU/m^3^ when the spread plate method was used to enumerate the bacterial colonies.

The minimum lethal aerosol dose of *B. anthracis* endospores for humans is highly uncertain. In the earlier studies, the medium lethal dose (LD_50_) was primarily estimated for nonhuman primates and was in the range of 2,500–55,000 spores (Inglesby et al. 2002). In the real threat situation in the USA, 2011, the investigation of the fatal case of bioterrorism-related inhalational anthrax in a 94-year-old female retiree in Oxford, CT, USA showed that environmental sampling ruled out her home or nearby areas as a source of the anthrax endospores (Griffith et al. 2003). The evidences gathered during outbreak investigation suggest that this woman was exposed through a cross-contaminated bulk mail letter. As a final conclusion, a statement was developed that the woman probably inhaled only a few spores and that was enough to result in her death. With use of mathematical modeling, Fennelly et al. (2004) have shown that from 2 to 836 airborne *B. anthracis* endospores may be sufficient to develop inhalational anthrax in humans. Therefore, the opportunity to detect *Bacillus* endospores at low concentrations in air is crucial to reduce the risk for human exposure to this harmful pathogen.

In summary, we conclude that microorganisms present in the ambient room air interfere with precise quantification of *Bacillus* endospores when they are aerosolized in relatively low concentrations and the growth of bacteria in culture media is used as the only analytical method. Additional work is needed to assess usefulness of other analytical methods, for example molecular techniques, to increase the detectability of *Bacillus* endospores in the ambient air samples collected using the Sartorius MD8 sampler.
